# Adding fuel to the fire: The lipid droplet and its associated proteins in cancer progression

**DOI:** 10.7150/ijbs.74902

**Published:** 2022-10-17

**Authors:** Wenqin Luo, Huan Wang, Liangliang Ren, Zeyi Lu, Qiming Zheng, Lifeng Ding, Haiyun Xie, Ruyue Wang, Chenhao Yu, Yudong Lin, Zhenwei Zhou, Liqun Xia, Gonghui Li

**Affiliations:** Department of Urology, Sir Run Run Shaw Hospital, Zhejiang University School of Medicine, Hangzhou, China.

**Keywords:** lipid droplet, cancer metabolism, perilipins, CIDEs, DGAT1, DGAT2, ATGL

## Abstract

A lipid droplet (LD) is an organelle that consists of a phospholipid monolayer and a neutral lipid core, with proteins embedded in or attached to its surface. Until recently, cancers had long been regarded as genetic disorders with the abnormal activation of oncogenes and inactivation of tumor suppressor genes before their quality of a metabolic disorder began to be recognized. The last decade has witnessed the recognition of several metabolic characteristics of cancer cells, among which one is the accumulation of lipid droplets; therefore, attention has been given to exploring the role of LDs in carcinomas. In addition, there has been a remarkable expansion in understanding the complexity of LD's function in cellular homeostasis, including but not limited to energy supply, endoplasmic reticulum (ER) stress and oxidative stress management, or lipotoxicity alleviation. Thus, lipid droplet-associated proteins, which to a great extent determine the dynamics of a lipid droplet, have attracted the interest of numerous cancer researchers and their potential as cancer diagnostic biomarkers and therapeutic targets has been affirmed by emerging evidence. In this review, we systematically summarize the critical role of LDs in cancer and then focus on four categories of lipid droplet-associated proteins having the most direct influence on LD biosynthesis (diacylglycerol acyltransferase 1 (DGAT1) and diacylglycerol acyltransferase 2 (DGAT2)), degradation (adipose triglyceride lipase (ATGL)), and two renowned protein families on the LD surface (perilipins and cell death-inducing DNA fragmentation factor alpha-like effectors (CIDEs)). In this way, we aim to highlight their important role in tumor progression and their potential in clinical applications.

## Introduction

Metabolic reprogramming, an established hallmark of cancer, has been identified as a requisite for its initiation and progression in past decades 1. The Warburg effect is the first metabolic characteristic of cancer cells that was brought forward in 1920, exhibiting their propensity for aerobic glycolysis in glucose utilization 2. The interconnection of different metabolic pathways determines the complexity of reprogrammed metabolism, and a comprehensive alternated metabolic network involving glucose, lipids and amino acids has been uncovered by subsequent researchers 3. Among these prominent metabolic features, deregulated lipid metabolism has attracted great research attention in recent years and is recognized as an effective tactic adopted by cancer cells to thrive in a changing microenvironment 4. The significant role of lipids in membrane synthesis, energy supply and cellular signal transduction in cancer cells has been gradually discovered 4. Phospholipids and glycolipids, together with sterols, constitute biological membranes, providing material to sustain the rapid proliferation of cancer cells 5. Lipid rafts, a cholesterol- and sphingolipid-enriched dynamic microdomain on cell membranes, are reorganized in cancer cells and have been demonstrated to play a vital role in cancer metastasis 6, 7. Triglycerides are mobilized to efficiently generate fatty acids, which can be sequentially utilized to provide energy, building blocks and antioxidants 5. In addition, fatty acids act as precursors of small signaling molecules such as prostaglandin E2 (PGE2) to promote cancer progression 8. This extensive participation of lipids in tumor progression reflects the dependence of cancer cells on reprogrammed lipid metabolism, thus representing it as a potential target for cancer therapy 9.

Together with the boost of de novo lipogenesis driven by overexpressed enzymes such as fatty-acid synthase (FASN) 10, enhanced lipid storage seems to be another common characteristic in multiple cancer cells 11-13, which usually refers to the accumulation of a lipid storing organelle, lipid droplets. Although first reported in 1890, LD has long been underappreciated by researchers 14. After a century of silencing, accompanied by a surge in research interest on the relationship between obesity, lipid metabolism and different diseases, LDs finally began to come under the light. 14 Studies have revealed its dynamic structure and intricate functions beyond lipid storage 15. Moreover, its significant role in the development of metabolic diseases began to be realized, and one of them is cancer 16, 17. Herein, we summarized the critical role of LDs in cancer progression and focused on several significant lipid droplet proteins that determine LD biosynthesis, maturation and degradation to discuss their potential as diagnostic and therapeutic targets in clinical implications.

## Lipid droplet (LD) structure and dynamics

As organelles functioning as a lipid repository, LDs are widely distributed in the cytoplasm. Different from other organelles enclosed in the phospholipid bilayer, lipid droplets consist of a phospholipid monolayer embedded with varied proteins and a hydrophobic core enriched with neutral lipid-like triacylglycerol and sterol esters 14.

**Figure 1:**
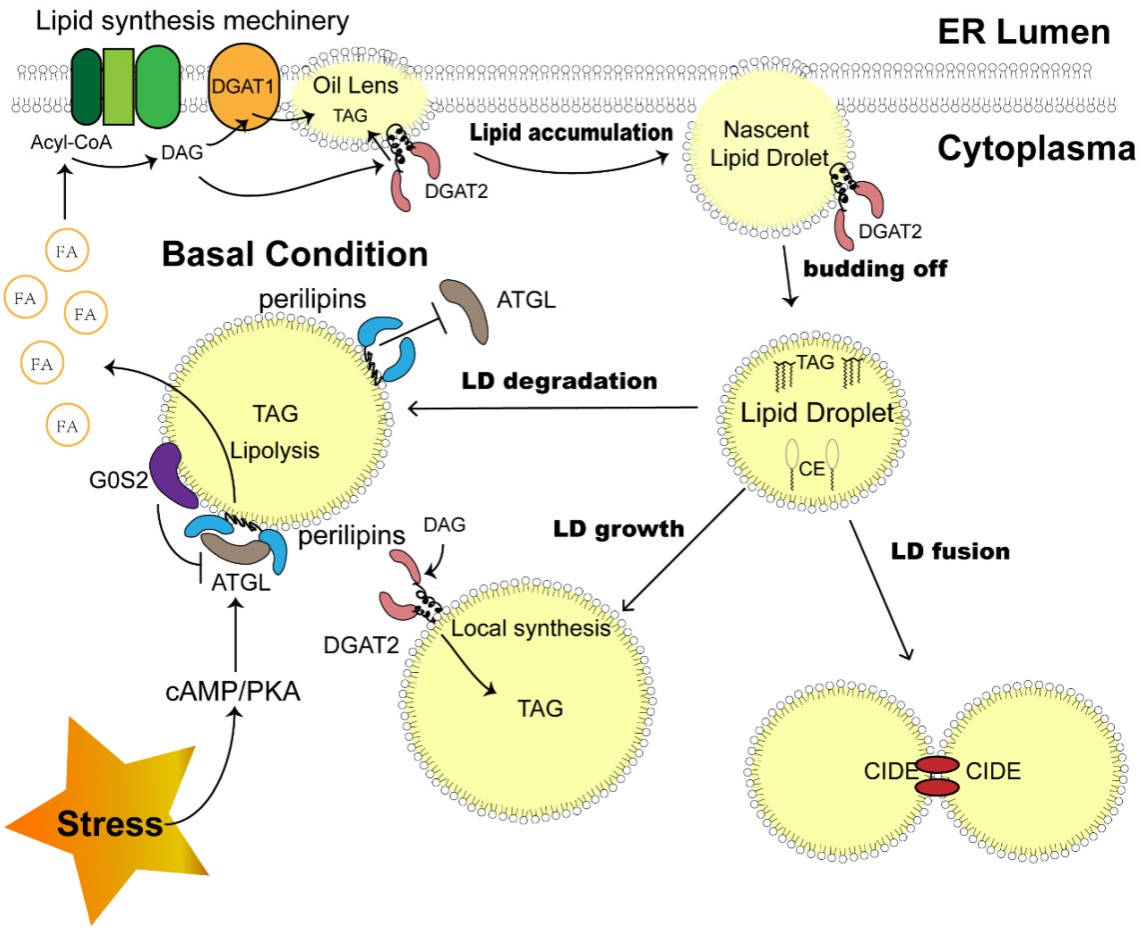
** Sketch of LD dynamics determined by LD-associated proteins.** LD biosynthesis is initiated with lipid accumulation between the two ER membrane leaflets. DGAT1 and DGAT2 catalyze the last step of TAG synthesis. After budding off, LDs expand through coalescence mediated by CIDEs and local TAG synthesis catalyzed by DGAT2 on their surface. A portion of ATGL is attached to the surface and triggers the first step of TAG hydrolysis, which could be impeded by perilipins on the LD surface under basal conditions. A stress-activated cAMP-PKA pathway phosphorylates perilipins and removes the block. G0S2 is another LD protein that inhibits TAG hydrolysis. **Abbreviations:** LD, lipid droplet; ER, endoplasmic reticulum; DGAT, diacylglycerol acyltransferase; TAG, triacylglycerol; CIDE, cell death-inducing DNA fragmentation factor alpha (DFFA)-like effector; ATGL, adipose tissue triacylglycerol lipase; cAMP, cyclic adenosine monophosphate; PKA, protein kinase A.

Although the mechanism of LD biogenesis has not been clarified, this process is recognized to occur in the endoplasmic reticulum (ER). The enzymes responsible for the esterification of fatty acids to generate sterol esters (SEs) and triacylglycerols (TAGs) reside on ER membranes, triggering the very first step of LD synthesis and lipid accumulation 15. Aggregating neutral lipids gradually form an oil lens between two leaflets of ER membranes, exhibiting the initial state of LD (Fig. 1).

Before LD becomes an independent unit, a budding-off process is needed. The discrepancy in membrane protein distribution and phospholipid constitution between the cytosolic and luminal sides of the ER membrane results in an imbalance in surface tension, which drives LD precursors to depart from the ER into the cytosol 18. The detailed mechanism of this process might be more complex, as other factors, such as seipin, were proven to be indispensable during LD budding off and were enriched beside the LD-ER membrane juncture 19, 20.

Investigations based on proteomics analysis revealed the presence of a variety of proteins in LDs. In LDs isolated from mammalian cells, 100-150 proteins can be reliably detected 21, including those associated with lipid synthesis and lipolysis, membrane trafficking, and protein degradation. These lipid droplet proteins determine the function and behavior of LDs after budding off (Fig. 1). The detached LDs further expand through coalescence induced by cell death-inducing DNA fragmentation factor alpha (DFFA)-like effector (CIDE) family proteins, and localized lipid synthesis takes place as the enzyme diacylglycerol acyltransferase 2 (DGAT2) translocates to the surface of LDs 22. The dynamic LDs in the cytoplasm ensure cellular metabolic balance 15. The degradation and sequential hydrolysis of lipids is a reliable mechanism employed by cells to fill the energy gap under metabolic stress, as the released free fatty acids (FFAs) would be efficiently utilized in mitochondria to produce ATP 5, 23. Under this condition, lipolysis enzymes such as adipose triglyceride lipase (ATGL), hormone sensitive lipase (HSL) and monoacylglycerol lipase (MGL) will be recruited to the surface of LDs to catalyze the reaction 24, which is mediated by hormone activating signal transduction 24, 25. In addition, perilipin family proteins also play a role in this process by coordinating the contact between lipids and lipases 26. Recently, lipophagy was also reported as a novel autophagosome-dependent process to decompose LDs 27, suggesting the much more intricate dynamics of LDs to sustain metabolic homeostasis.

## Lipid droplets in cancer progression

**Figure 2:**
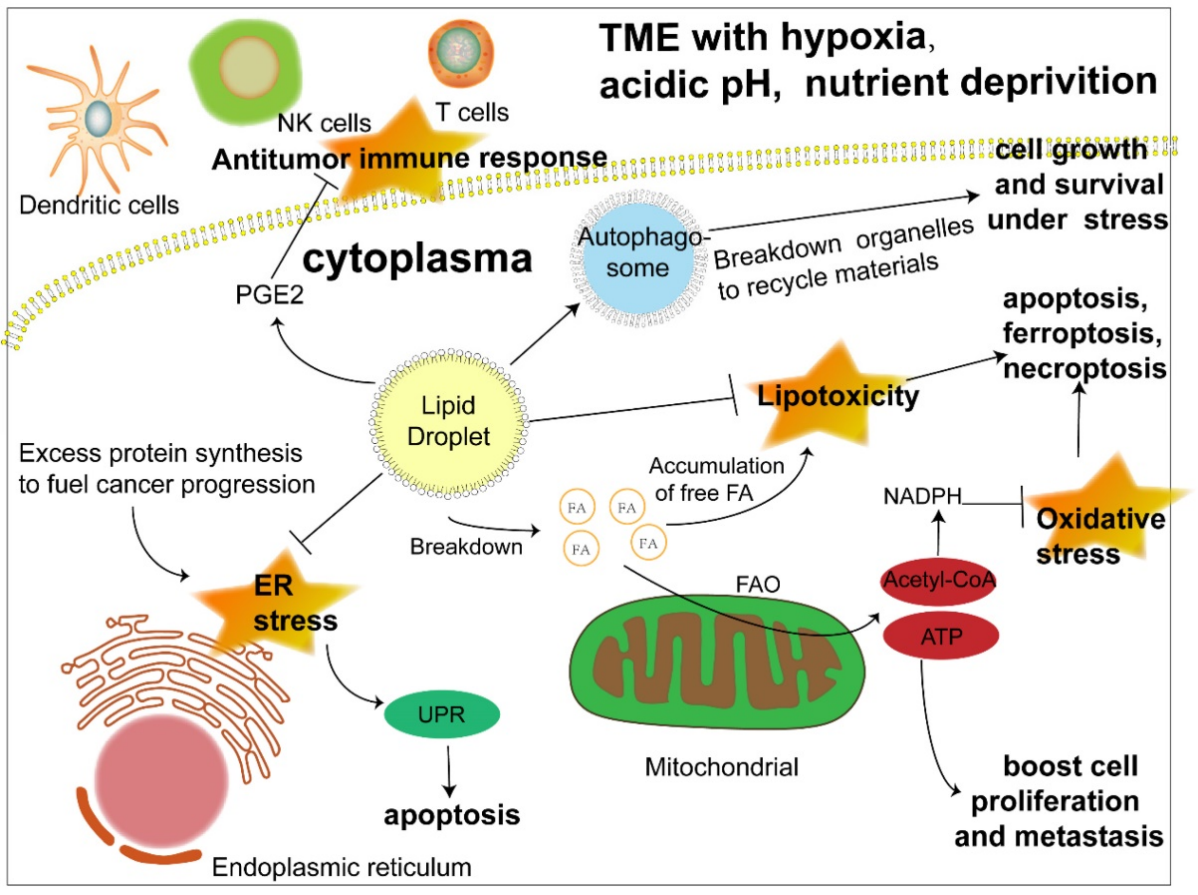
** The significant role of LDs in cancer progression under stress.** Lipid droplets function in multiple ways to promote cancer progression. 1) LDs release fatty acids to generate acyl-CoA and channel them to mitochondria to produce energy through FAO to boost cancer cell proliferation and metastasis. 2) Another product of FAO, acetyl-CoA, can produce NADPH, which acts as a hydrogen donor to maintain redox homeostasis and prevent cell death induced by excessive ROS accumulation. 3) LDs play an important role in ameliorating ER stress caused by abundant newly synthesized unfolded proteins in cancer cells, otherwise triggering apoptosis. 4) LDs sequester detrimental lipids such as DAG, cholesterol, ceramide and polyunsaturated FAs that tend to be oxidized into their core to prevent apoptosis, ferroptosis and necroptosis caused by lipotoxicity. 5) LD initiates autophagy by promoting the formation of autophagosomes to recycle materials from destroyed organelles in cancer cells under metabolic stress. 6) LD stores the precursor to produce signaling molecules such as PGE2, which could interfere with the antitumor immune response. **Abbreviations:** ATP, adenosine triphosphate; ER, endoplasmic reticulum; FA, fatty acid; FAO, fatty acid oxidation; NADPH, nicotinamide adenine dinucleotide phosphate; NK cell, natural killer cell; PGE2, prostaglandin E2; UPR, unfolded protein response.

The biosynthesis and degradation of lipid droplets, as a pivotal part of lipid metabolism, is integral to the maintenance of normal cellular physiology and is naturally considered to be linked to disease progression, especially metabolic disorders. Previous studies have revealed its relationship with diabetes, fatty liver disease and atherosclerosis 16,17,28. Much more recently, the metabolic characteristics of cancer have been a research focus and viewed as prospective therapeutic targets, among which abnormal lipid accumulation in cancer cells is a salient one. Moreover, investigations are beginning to shed light on the role of LDs, the lipid content depot, in the development of cancer (Fig. 2).

Confronted with a tumor microenvironment (TME) that is changing rapidly and sometimes inhospitable due to oxygen and glucose deprivation as well as acidosis, cancer cells depend on lipid mobilization to survive 5, 29. The FA esterified as TAG and SE in lipid droplets can be released to generate acyl-CoA and provide energy by β-oxidation to fuel cancer cell proliferation and metastasis 30. In addition, closer contact between LDs and mitochondria was also revealed 31, 32. Hermes et al. reported a stronger interaction between LDs and mitochondria activated by AMPK signaling under nutrient starvation due to the translocation of both organelles from the cell center to the periphery along with a subset of microtubules, hence a more effective utilization of FAs to fill the energy gap 31. Consistently, an earlier study proposed an intricate mechanism of channeling FAs from LDs to mitochondria under starvation, which was associated with cytoplasmic lipases, autophagy and the fusion of adjacent mitochondria 32. However, more research on the interaction between LDs and mitochondria is still required to gain a comprehensive understanding of LD function in lipid utilization. Apart from energy, cancer cells also require membrane expansion to sustain their fast proliferation and growth, which further highlights the importance of triacylglycerol and cholesteryl esters stored in LDs as a lipid source of nascent membranes 33. Despite cancer cell dependence on FAs as an energy source, when lipids are not urgently needed, their excess accumulation leads to lipotoxicity 34, 35. The formation of LDs is beneficial to the survival of cancer cells by isolating lipids such as ceramides and DAG that can induce inflammation 36 and protecting polyunsaturated FAs from lipid peroxidation, which could sequentially result in DNA damage and cell death 37. All of the above results reflect the dynamism of LDs as a flexible machinery adopted by cancer cells to cope with unstable surroundings and efficiently meet their energy demand.

Another crucial function of LDs is protecting cancer cells under ER stress and oxidative stress. The ER is an organelle responsible for the maturation of proteins owing to its capacity for protein folding 38, which, however, is not unlimited 39. Thus, ER stress occurs when abundant proteins are synthesized in a short time, which is common in cancer cells 39. Even though cells initiate the unfolded protein response (UPR) to cope, irreversible damage and the induction of apoptosis are still inevitable when ER stress is overwhelming. LDs are considered to participate in the management of ER stress, possibly by regulating fatty acid composition in the ER membrane 40 and removing misfolded proteins 41, although further investigations are required to elucidate the detailed mechanism. The increase of reactive oxygen species (ROS) is a characteristic of cancer cells. While they can activate protumorigenic signaling pathways, excess ROS can trigger apoptosis, necroptosis and ferroptosis 42. Antioxidants such as NADPH are crucial to alleviate oxidative stress and sustain ROS homeostasis 43. Acetyl-CoA, the main product of FAO, enters tricarboxylic acid cycle (TCA) and generate citrate 44, which could be transformed into isocitrate and malate to synthesize NADPH, with the catalyzation of isocitrate dehydrogenase 1 (IDH1) and malic enzyme 1 (ME1) respectively 45-47. When the NADPH producing by pentose phosphate pathway is inhibited for a glucose deprived TME, the two forementioned pathways driven by FAO became vital to eliminate the possible detriment brought by these reduction products of oxygen in cancer cells 45, 48.

Moreover, increasing evidence has suggested an even more extensive role of LDs in cancer progression. Autophagy is a self-degradative process that maintains intercellular energy balance by breaking down damaged or senescent organelles and recycling their materials 49, an important mechanism adopted by cancer cells to cope with nutrient stress. While LDs have long been regarded as targets of autophagy, researchers have found that LDs are vital to initiate this process, which might be attributed to providing lipids to form autophagosomes and the maintenance of an appropriate phospholipid composition 50, 51. Reprogramming metabolism is closely linked to the antitumor immune response, and LDs are involved in this connection. It has been demonstrated that LDs are the sites for the synthesis of prostaglandin E2, an established suppressor of antitumor immunity 52, 53. In addition, LDs were reported to influence the function of immune cells such as dendritic cells and T cells 13, 54. Although the exploration of LDs in cancer immunity is still in its infancy, it is safe to assume that LDs are a potential target to modulate antitumor immunity.

## Lipid droplet-associated proteins and their role in cancer

As mentioned above, emerging evidence has demonstrated the versatility of LDs in cancer progression, which promotes the thriving of cancer cells in a volatile and unfriendly environment. 4, 55 While LDs function as organelles, their biosynthesis, degradation and other behaviors essentially depend on their associated proteins 15. Naturally, a great number of studies have focused on these proteins and revealed their differential expression in specific cancer types or their correlation with tumor stages and outcomes, suggesting that these lipid droplet proteins have diagnostic and prognostic value in clinical practice 56-58. Furthermore, functional experiments both *in vitro* and *in vivo* further revealed their impact on cancer phenotypes such as proliferation, metastasis and chemotherapy resistance, implying their potential as therapeutic targets to interfere with homeostasis in cancer cells 12, 59-61 (Table 1). LD proteomics studies have revealed the existence of over 100 kinds of proteins on LD surfaces, some of which are enzymes involved in lipid metabolism, while others consist of histones, ribosomal proteins, and particular LD-residing proteins 21. In this review, we focus on the proteins most directly impacting LD biosynthesis (DGAT1 and DGAT2) and degradation (ATGL) and two noted protein families enriched in the phospholipid monolayer of LDs (perilipins and CIDEs) to summarize their important role in tumor progression and outline their potential as diagnostic and therapeutic targets.

### Perilipin family

Perilipins are a family of proteins detected on the surface of LDs. In mammalian cells, PLIN1-5 were identified to play an important role in sustaining lipid homeostasis, setting a balance between lipid storage and utilization 62. All of these members share two conserved motifs in their N-terminal: a PAT domain and a repeating 11-mer helical motif, while heterogeneous structures are exhibited in their C-terminal 26. It has been assumed that the conserved N-termini anchor these proteins to the surface of a lipid droplet, but subsequent research revealed that the C-termini were also indispensable 63, 64. As some of the best-studied proteins reside on LDs, PLINs were found not only to be engaged in the biogenesis of lipid droplets 65, 66 but also to play a vital role in regulating both classical lipolysis 67, 68 and autophagic lipolysis 69, 70. Their roles in traditional metabolic diseases such as fatty liver disease, type 2 disease and obesity have long been recognized. Recently, with cancer metabolism becoming a highly focused area, as proteins that determine the behaviors of LDs, the influence of PLINs in cancer progression was gradually uncovered.

#### PLIN2 and PLIN3

In contrast to the other three perilipin family members that appear in specific tissues, PLIN2 (also known as adipose differentiation-related protein, ADRP) and PLIN3 (known as TIP-47) are more ubiquitous 71, contributing to the formation of LDs in various cell types. The expression of PLIN2 is regarded as a marker reflecting the lipid content of cells 71. Studies have revealed the involvement of PLIN2 in the regulation of cytosolic lipolysis by interfering with ATGL access to lipid droplets 72. Meanwhile, unlike PLIN1 and PLIN5, whose direct binding with lipase has been identified, PLIN2 has not been found to interact with ATGL, which might account for its relatively mild effect in this classical process of lipolysis. Interestingly, PLIN2 was also shown to act as a barrier blocking another new autophagy-related approach of fat mobilization, named macroautophagy 73. PLIN3 has been revealed to be decorating the coat of newly synthesized lipid droplets 74, and both *in vivo* and *in vitro* studies suggested that it protects lipid droplets from lipolysis, similar to PLIN2 75, 76. Another study reported that both PLIN2 and PLIN3 are chaperone-mediated autophagy targets whose removal could promote LD mobilization under nutrient stress by enhancing both macroautophagy and classical lipolysis 77. Recently, Lu et al. reported that CHKα2 phosphorylates PLIN2 and PLIN3 to facilitate lipid droplet degradation and β oxidation 78.

Renal cell carcinoma is one of the most commonly diagnosed cancers worldwide, and its incidence rate has increased in recent years. Although the application of targeted drugs such as sunitinib, pazopanib and novel immune checkpoint inhibitors brought hope to patients with advanced clear cell renal cell carcinoma (ccRCC), its prognosis remains poor 79. The accumulation of LDs has been recognized as a noticeable hallmark of ccRCC, and PLIN2 has been well studied in ccRCC to explore its biological role in cancer progression and its potential clinical application. Masahiro Yao et al. detected considerably increased expression of PLIN2 in renal cell carcinoma tissues 80. To validate the possibility of urine PLIN2 as an early diagnosis indicator, a prospective case‒control study adopted patients with incidental radiographically discovered renal masses and presurgical presumptive diagnoses of kidney cancer, where patients who underwent other surgeries and healthy volunteers acted as controls 81. It turned out that urine PLIN2 concentration in patients diagnosed with ccRCC or papillary cancer greatly exceeded that of the control group. Subsequent clinical research verified the specificity of urine PLIN2 to differentiate clear cell or papillary kidney cancer from other nontumorous kidney diseases 82 and other urological cancers 83.

Given the evidence from clinical studies, some studies have begun to explore the molecular mechanism of PLIN2 in renal cell carcinoma. The inactivated mutation of the Von Hippel-Lindau (VHL) gene has been identified as a key mutation in ccRCC that facilitates cancer progression 84. The absence of its product, pVHL, leads to an abnormal accumulation of the oncogenic transcription factor HIF2α, which is strengthened by hypoxia 85. A recent study revealed that PLIN2 expression was also upregulated by HIF-2α, subsequently promoting lipid storage 12. The author revealed that the elevated expression of PLIN2 preserved the viability of ccRCC cells by decreasing UPR triggered by extra protein synthesis and maintaining ER homeostasis in cancer cells.

Irregular lipid accumulation is also a characteristic of prostate cancer (Pca) cells, another urological cancer with a high incidence in old males. Yue et al. identified that PTEN loss driving the accumulation of esterified cholesterol in LDs was notable in advanced prostate cancer tissue. The inhibition of cholesterol esterification substantially suppressed Pca growth and aggressiveness by downregulating the uptake of LDL 11. Other studies revealed that the dysregulation of lipid metabolism was directly related to the development of resistance to first-line ADT treatment in prostate cancer, partly attributed to PLIN2-containing exomes released by the cells treated with ADTs in a paracrine manner 86, 87, 88. Another study identified the role of PLIN3 in Pca, protecting cells from apoptosis induced by ER stress. Moreover, knockdown of PLIN3 was found to decrease the intratumoral synthesis of androgen and restore the ADT treatment sensitivity of enzalutamide-resistant C4-2 cells. These works collectively suggest that targeting LDs and perilipins is promising for the treatment of CRPC patients 59.

In addition to ccRCC and PCa, the involvement of PLIN2 in other cancers has also been detected. A pathological study found that PLIN2-positive lung adenocarcinoma was associated with a worse outcome reflected in overall and disease-free survival 89. This was supported by basic research indicating that PLIN2 can promote lung adenocarcinoma cell proliferation by increasing the phosphorylation of Akt 90. Kimberly et al. reported that PLIN2 possesses potential in breast cancer subtype differentiation, as high-grade subtypes, namely, Her2-positive and TNBC, have lower expression of this protein 91. In addition, a better clinical outcome, reflected in the recurrence-free survival time in the PLIN2 low expression group, was presented. Another study conducting plasma proteome analysis found elevated expression of PLIN2 in colorectal cancer patient plasma, even in those with low-grade tumors, indicating that PLIN2 is a sensitive and dependable biomarker in this cancer type 92.

Studies focusing on PLIN3 have been less frequent; however, its value in cancer diagnosis and treatment is still worth exploring. Immunohistochemical analyses in a large number of specimens of multiple cancers demonstrated a positive correlation between PLIN3 staining intensity and tumor size in colorectal cancer, as well as tumor grade and stage in lung cancer 93. In cervical cancer, PLIN3 was found to be elevated in patients with invasive tumors, lymph nodular metastasis, and recurrence after treatment 94.

#### PLIN1, PLIN4 & PLIN5

The distribution of these three perilipins is tissue specific. As the first identified protein in this family, PLIN1 (known as perilipin) occurs mainly in the adipocytes of white and brown adipose tissue, where the lipid droplets are relatively large. Many researchers focused on its role in lipid mobilization, and they found that the cAMP-PKA pathway activated by epinephrine and norepinephrine greatly phosphorylated PLIN1 in adipocytes under fasting or excise conditions, which sequentially recruited ATGL and HSL to separately hydrolyze TAG or DAG 95. A delicate scaffolding complex is in charge of this efficient switch when extra energy is needed, which consists of PLIN1 and αβ hydrolase domain containing 5 (ABHD5), an activator of ATGL, on the surface of LDs in white adipocytes 96. It was demonstrated that PLIN1 possesses prognostic value and therapeutic potential to suppress cell proliferation both in breast cancer and liposarcoma 57, 97-99. Ronell et al. reported that PLIN1 was also upregulated in conventional ameloblastoma and ameloblastic carcinoma, two kinds of odontogenic tumors, compared with tooth germ tissue 100.

PLIN4, the largest member of this family, is mainly located in LDs in brown adipose tissue, skeletal muscle and the heart. It appears on some nascent LDs or remains unanchored 74. Chen et al. found a decrease in TAG content in the hearts of PLIN4(-/-) mice 101. Another recent study disclosed that it functions to decrease the size of LDs, relying on its much longer amphipathic helices compared with other perilipin members 102. The exploration of its specific functions on lipid droplets is still in its infancy, while some studies revealed that Plin4 might have a role in cancer progression. A research group focusing on chemoresistance in triple-negative breast cancer (TNBC) demonstrated that PLIN4 was overexpressed in doxorubicin-resistant cells and could be targeted to eliminate chemoresistance 103. Li et al. also revealed its value as a diagnostic biomarker of liposarcoma and could refer to the discrimination of its subtypes 97.

Similar to PLIN4, PLIN5 is distributed in oxidative tissue and is indispensable for maintaining lipid storage and modulating lipolysis in this tissue 26, 104. It can bind ATGL and HSL to prevent TG hydrolysis, and similar to PLIN1, it is phosphorylated by PKA for its control to be sequentially lifted 104. Similar to other members of the perilipin family, some evidence of its role in cancer has been uncovered. Combined with other perilipin members, it can serve as a diagnostic marker in breast cancer 57. Researchers also detected an increase in the expression of PLIN5 in a murine HCC model and clinical specimens 105.

All the above publications suggest that PLIN1, PLIN4 and PLIN5 can be involved in cancer progression, despite their tendency to distribute in adipocytes. As paracancerous adipose tissue was identified as an essential part of the TME to influence the proliferation, migration and drug resistance of cancer cells, subsequent studies will shed light on how these perilipin members act in cancer progression.

### CIDE (cell death-inducing DNA fragmentation factor alpha (DFFA)-like effector) family

CIDE family proteins, as their name implies, are characterized by their cell death-inducing DFF45-like effect domain in their N-terminus. Three members have been identified in humans: CIDE-A, which is mainly expressed in WAT and the mammary gland, CIDE-B, which is mainly present in the liver, and CIDE-C (also known as FSP27), which is mainly expressed in WAT and BAT 106. Previous studies focused on their apoptosis-inducing effect, but the research interest in this protein family shifted as they were also found to reside on lipid droplets. These proteins were demonstrated to play an important role in promoting lipid accumulation by mediating LD fusion, since a smaller LD surface area/volume ratio reduces the accessibility of lipases to lipids 107, 108, 109. Enriching in the LD-LD contact sites, CIDE proteins from two LDs interact with each other to form a channel to enable lipid transfer coordinated by two other lipid droplet-associated proteins, PLIN1 and RAB8 110, 111.

The dual role played by CIDE family proteins makes it more challenging to elucidate their effect on cancer progression. Li et al. reported that CIDE-C suppressed the proliferation of cancer cells by triggering apoptosis; therefore, it could be a therapeutic target in carcinomas due to the high-frequency mutation in its chromosome region 112. One study on hepatocellular carcinoma exhibited the downregulation of CIDE‑C in HCC tissues compared with adjacent normal tissue, as well as an obvious proapoptotic effect caused by the overexpression of CIDE-C in HCC cells, which could be explained by the interaction between CIDE‑C and lipopolysaccharide-induced tumor necrosis factor (LITAF) 113. Another functional study revealed that this proapoptotic effect in HeLa cells could be greatly mitigated when supplementary oleic acid was added to the medium. The author inferred that CIDE-C tends to be located on LDs after extra FA supply and that subcellular distribution alteration may prevent it from activating the apoptosis process 114. In clear cell renal cell carcinoma (ccRCC) characterized by remarkable LD accumulation, it was demonstrated that CIDE-C was upregulated in cancer as expected, but CIDE-B was downregulated. The expression of CIDE-B was also lower in high-grade ccRCC than in low-grade ccRCC, and this was associated with a better prognosis 56. In summary, earlier studies explored the role of CIDE in cancer based on its apoptosis-inducing effect. Since its new function in LD dynamics was identified, more research was prompted to comprehensively illustrate the linkage between CIDE family members and cancer progression in the context of cancer metabolic reprogramming.

### DGAT1 & DGAT2

During the process of lipid droplet formation, diacylglycerol acyltransferase (DGAT) enzymes catalyze the last step of TG synthesis from fatty acyl-CoA and diacylglycerol (DG). DGAT1 and DGAT2, although responsible for the same biochemical reaction, are encoded by genes located in different positions on chromosomes 115, which brings about a distinction in the structures of these two proteins. Their subcellular locations revealed by fluorescence imaging have shown that DGAT1 appears exclusively on ER membranes, whereas DGAT2 resides both on ER membranes and in lipid droplet phospholipid monolayers 116. An LD target domain in DGAT2 originally embedded in the ER membrane was proven necessary for its targeting on LDs, facilitating the budding-off process to form nascent LDs after the regional accumulation of TGs 117. It was reported that DGAT2 promotes the expansion of LDs by synthesizing TG at its surface 22. These two enzymes also present different propensities to their substrates; the catalytic activity of DGAT1 is more concentrated on lipogenesis with exogenous FA, while DGAT2 does not discriminate the FA source 115, 118. Reflecting this distinction in their tissue distribution, DGAT1 is ubiquitous but particularly highly expressed in intestine and adipose tissue, whereas DGAT2 is mainly expressed in hepatocytes and adipocytes 119.

Glioblastoma (GLB) is recognized as a malignant brain tumor with the hallmark of abundant LDs. Guo et al. revealed that GLB is reliant on the upregulation of DGAT1 but not DGAT2 to synthesize TG and form LDs 61. Targeting DGAT1 in GLB leads to ROS accumulation by consequently intensifying the β-oxidation of free fatty acids. Furthermore, ROS impair the oxidative ability of mitochondria, sequentially resulting in the accumulation of acetyl-CoA, which augments oxidative stress and finally triggers apoptosis. In prostate cancer, it was demonstrated by Crawford et al. that the expression of DGAT1 increased in cancer tissues, and cell proliferation and migration could be inhibited by targeting DGAT1 120. Another recent study discovered that the upregulation of DGAT1 in prostate cancer is partly mediated by EPH receptor B2 (EPHB2), a tyrosine kinase ephrin receptor 121. Studies on ovarian cancer and breast cancer also demonstrated the potential of DGAT1 as a novel target to repress cancer progression 58, 122. The featured tumor microenvironment was considered to drive metabolic reprogramming in cancer cells. Feron et al. first reported that acidosis, a characteristic of the TME, leads to LD accumulation in cancer cells by upregulating DGAT1 and CD36, mediated by TGF-β signaling. Moreover, the authors found that LD accumulation supports cancer metastasis by abating anoikis 123.

DGAT2 deserves as much attention as DGAT1, if not more, in cancer research. A study focusing on gastric cancer revealed increased expression of DGAT2 in cancer cells cocultured with adipocytes. Silencing DGAT2 made these cells vulnerable to anoikis and efficiently inhibited cancer metastasis *in vivo* and *in vitro* 124, suggesting that it is a promising target in patients with GC. In the MCF-7 breast cancer cell line, Seco et al. found that the pharmacological inhibition of DGAT2 efficiently restrained their migration and increased their sensitivity to radiation treatment 125. However, in some other cancers, such as hepatocellular carcinoma 126 and melanoma 127, DGAT2 was identified to play a suppressive role. Shen et al. revealed that DGAT2 is downregulated in HCC tissue, and its low expression implies a shorter survival time 126. Overexpression of DGAT resulted in evidently inhibited proliferation in HCC cell lines, with decreased expression of cell cycle-related genes 126, suggesting that DGAT2, as a key protein in modulating lipid metabolism, may also function in other aspects.

### ATGL

Adipose triglyceride lipase (ATGL) was identified as a rate-limiting enzyme in lipolysis and in charge of the very first step of TG hydrolysis by Zimmermann, R. et al. in 2004 128. It is highly expressed in adipose tissue but can also be detected in liver, heart and skeletal muscle. Subsequent research reported a massive amount of fat accumulation in adipocytes and muscle in mice induced by the lack of ATGL, which suggests its vital role in lipid metabolism 129. By exploring its subcellular location in cells, researchers detected part of ATGL on the surface of LDs, as the majority of ATGL is dispersedly distributed in the cytoplasm 128. This finding indicates that LDs provide a platform for lipid hydrolysis. Later, studies confirmed this conjecture and uncovered an intricate interaction mode between ATGL and other lipid droplet-associated proteins to modulate lipid metabolism 95, 130. Under basal conditions, ATGL can be bound and activated by ABHD5, another protein on LDs, to moderately perform hydrolysis 131. As mentioned previously, Perilipin family proteins impede ATGL's access to TAG in LDs by separating it from its coactivator, ABHD5. This interference ceases during fasting due to cAMP-PKA pathway-mediated phosphorylation. Liu et al. revealed that G0S2 also acts to inhibit ATGL's lipase activity 132. Another protein, hypoxia-inducible gene 2 (HIG2), which is activated by HIF-1, was validated to inhibit ATGL and consequently promote lipid accumulation in hypoxia 133.

Even though it has been just over a decade since the mechanism of ATGL was clarified 128, growing evidence is establishing its vital role in cancer, given that it is closely related to lipid synthesis and fatty acid oxidation (FAO), two rewired pathways in cancer cells 4, 30. As abundant lipid synthesis and LD accumulation are metabolic hallmarks of multiple cancers, ATGL, as a lipase driving the reverse reaction, was naturally revealed as a suppressor of malignancy 134, 135. In multiple mouth cancer cell lines, ectopic overexpression of ATGL repressed cell proliferation 130, 136. Liu et al. declared the importance of ATGL downregulation in cancer cells to survive in hypoxia, a characteristic of the TME 133. A study in hepatocellular cancer verified that ATGL, which is significantly downregulated in HCC tissues, will switch cancer cells' preference for an energy source from glucose to FFA and inhibit cell proliferation. Moreover, the recognized tumor suppressor p53 was identified as a possible downstream target of ATGL 135. Similar phenomena were observed for ATGL in ovarian cancer 137. Meanwhile, its effect in lung adenocarcinoma remains somewhat elusive. Earlier, *in vitro* research proposed a pro-neoplasm role 138. However, it seems that an opposite opinion prevails since Hoefler et al. revealed its reduction in tumor tissue and that knockout of ATGL in mice greatly enhanced their risk of having pulmonary neoplasm 139. Another study adopting a 3D *in vitro* model found that ATGL knockout promoted the growth of spheroids and facilitated cancer cell adaptation to hypoxia 133. Furthermore, the depletion of ATGL was validated to present a more aggressive phenotype, attributed to the consequent upregulation of SRC kinase signaling 140. Beyond its functions in cancer cells, a new discovery shed light on ATGL's role in the crosstalk between cancer cells and the TME. Li et al. reported repressed expression of ATGL in neutrophils, which was induced by its interaction with resident mesenchymal cells, contributing to breast cancer colonization in the lung. Moreover, neutrophils sequentially transfer stored lipids to cancer cells to enable their survival and proliferation 60. These results exhibit promising prospects to interfere with the metabolic rewiring of cancer cells by targeting ATGL.

However, in certain carcinomas, this lipase was recognized as an oncoprotein; it is reasonable to consider that many malignancies present greater reliance on FAO to fuel their progression. The increased utilization of TG in LDs through overexpression of ATGL was demonstrated to promote the migration and proliferation of HeLa cells, paralleled by activated HIF-1a-mediated mitophagy to mitigate potential damage caused by ROS accumulation 141. Obesity has long been regarded as a risk factor in breast cancer. Obtaining free fatty acids from adipocytes in a coculture system, breast cancer cells showed an increase in proliferation and migration activity, which was dependent on ATGL inducing lipolysis both in adipocytes and cancer cells 142, 143. Iftikhar et al. reported that ATGL might play a vital role in the development of colon cancer driven by obesity. *In vitro*, the increase in proliferation induced by oleic acid was eliminated through the inhibition of ATGL. Except for obvious lipid storage, some established oncogenes, such as RARA or MYC, were also found to be decreased after ATGL knockdown 144.

All these results imply that the role of ATGL in cancer is rather complex and not limited to consuming TG in LDs but also linked to other pathways. In addition to its nonenergetic functions that have begun to be clarified 145, 146, more studies are required to comprehensively address its position in cancer progression before its clinical potential can be fully realized.

## Conclusions and Perspectives

Lipid droplets, as cytoplasmic organelles that were previously regarded as a pure depot of neutral lipids, are now recognized to have impacts on cells in multiple ways. Its dynamics (biosynthesis, expansion and degradation) are considered a significant indication of the metabolic status of cells in different biological contexts. Therefore, scientists employed diverse fluorescent imaging techniques to detect and observe LDs in living cells 147, 148, which enhanced the understanding of their vital role in cell metabolism and presented their connection with the development of metabolic disorders such as obesity, type 2 diabetes, cardiovascular disease, and cancer. However, unanswered questions still remain. Multiple studies have revealed the links between LDs and other cellular organelles, such as mitochondria, ER, and lysosomes, which permit material exchange and influence the trafficking of these organelles 31, 149, 150. Nonetheless, the detailed functions and mechanism of this relationship remain elusive; thus, further studies are needed to elucidate the potential synergistic mechanism of these organelles and their impact on cellular physiology and pathological processes. In cancer research, another alluring question is how LDs mediate the crosstalk between cancer cells and their TME consisting of adipocytes, cancer-associated fibroblasts, immune cells, and so on. While some communications dependent on lipid metabolism have been deciphered 13, 124, a more comprehensive picture is awaiting construction, which will be possible by virtue of the maturation and popularization of single-cell sequencing techniques.

Great progress has been made to study lipid droplets in basic oncology research; however, their potential as clinical diagnostic and prognostic indicators has failed to be fully realized due to the unfeasibility of detection and the lack of generally applicable quantification methods. The proteins residing on LDs could be an alternative to overcome this challenge. In fact, the dynamics of LDs could be reflected by the expression of LD-associated proteins, as the behaviors and functions of LDs greatly depend on these proteins. Numerous studies have claimed the differential expression of lipid droplet-associated proteins in various neoplasms and their correlation with tumor stages and prognosis. Moreover, some studies reported that these proteins could be detected in body fluids such as serum or urine 83, 94, indicating their potential as noninvasive biomarkers in the clinic. Notwithstanding, studies involving large cohorts are required before these proteins can be practically used to guide cancer diagnosis and treatment.

Recently, targeting lipid metabolism in cancer cells has been explored as an anticancer strategy, given the pivotal role of reprogrammed lipid metabolism in tumor initiation and progression. For instance, FASN, the enzyme that functions to catalyze *de novo* lipogenesis, is being established as an applicable and promising therapeutic target, with its inhibitor TVB-2640 going through clinical trials in a variety of cancers, including NSCLC, CRC, HER2+ advanced breast cancer, and astrocytoma. Fatty acid uptake, namely, CD36, represents another target, and its inhibitor ABT-50 has also entered clinical trials in melanoma 151. As another significant constituent of lipid metabolism, the storage and mobilization of intercellular lipids and their critical organelles, lipid droplets have not yet received the attention they deserve. However, cancer cell plasticity in lipid uptake, acquisition and utilization, partly owing to the mobilization of lipids stored in LDs, brings resistance to treatments targeting a single pathway. A combinational therapy with multiple targets in lipid metabolism might be conducive to disrupting lipid homeostasis in cancer cells; therefore, there is still room for LD-associated proteins in cancer treatment. To this end, as highlighted in this review, lipid droplet-associated proteins participate in cancer reprogrammed metabolism and influence tumor progression in diverse aspects, which substantiates the claims that exploring their potential as therapeutic targets is needed to expand the options for clinical cancer treatment. Looking forward to future research, with the growing understanding of LD-associated proteins and the evidence presented by large cohort studies and clinical trials expanding, we envisage that these proteins, representing one of the most intriguing metabolic characteristics of cancer cells, are to be adopted both as viable cancer biomarkers and effective oncotherapy targets.

## Figures and Tables

**Table 1:** Impacts and clinical implications of LD-associated proteins in various cancers

Tumor type	LD associated proteins	Expression	Outcomes	Reference
Renal cell carcinoma	PLIN2	Up	Promotes cell proliferation; High expression indicates good prognosis.	12, 81, 83, 152
	PLIN3	Up	Decreases sunitinib sensitivity; High expression indicates poor prognosis.	153, 154
	CIDEB	Up	High expression indicates good prognosis.	106
Prostate cancer	PLIN3	-	Promotes cell proliferation; Increase resistance to radiation therapy. High expression indicates poor prognosis.	59, 155
	DGAT1	Up	Promotes cell proliferation.	156
Breast cancer	PLIN1	Down	Reduces cell migration; High expression indicates good prognosis.	57, 98
	PLIN2	Down	Elevated in HER-2 positive subtype; High expression indicates poor prognosis.	57
	PLIN4	Down	Elevated in doxorubicin resistant cells; High expression indicates poor prognosis in patients receive chemotherapy.	57, 103
	DGAT2	-	Promotes cell migration, increase resistance to radiation therapy. High expression indicates poor prognosis in HER-2 patients.	125, 157
	ATGL	Up	Promotes cell proliferation and invasion; Elevated in HER-2 subtype; High expression indicates poor prognosis.	142, 143
Hepatocellular carcinoma	PLIN5	Up	-	105
	CIDE-C	Down	Induces cell apoptosis.	113
	DGAT2	Down	Reduces cell proliferation; High expression indicates good prognosis.	126
	ATGL	Down	Reduces cell proliferation	135
Gastric cancer	PLIN2	Up	Promotes cell proliferation and reduce cell apoptosis and ferroptosis.	158
	DGAT2	-	Promotes cell anoikis resistance and promote metastasis; Elevated in metastatic tumor tissue; High expression indicates poor prognosis.	124
	ATGL	-	High expression indicates good prognosis.	139
Colorectal cancer	PLIN2	Up	Elevated significantly even in early stage.	92
	ATGL	Up	Promotes cell proliferation.	144
Ovarian cancer	DGAT1	-	Promote cell proliferation, tumor colonization and metastasis; Elevated in patients with venous invasion; High expression indicates poor prognosis.	58
	ATGL	-	Reduces cell proliferation, migration and invasion.	137
Cervical cancer	PLIN3	Up	Elevated in reoccurrence; High expression indicates poor prognosis.	94
	ATGL	-	Promotes cell proliferation, migration and invasion; High expression indicates poor prognosis.	141
Glioblastoma	CIDEA	Down	Reduces cell proliferation and induce apoptosis.	159
	DGAT1	Up	Eliminates ROS and reduce cell apoptosis; High expression indicates poor prognosis.	61
Lung cancer	PLIN2	Up	Promotes cell proliferation; High expression indicates poor prognosis.	89, 90
	DGAT1	Up	High expression indicates good prognosis.	160
	ATGL	Down	Reduces cell proliferation, migration and invasion; Induces cell apoptosis; High expression indicates good prognosis.	139, 161, 162
Liposarcoma	PLIN1	Up	Promotes proliferation and migration; Reduce cell apoptosis; Elevated in liposarcoma but absent in other sarcoma subtypes.	97, 99
	PLIN4	-	Elevated in liposarcoma but absent in other sarcoma subtypes.	97

## Abbreviations

LD: lipid droplet
ER: endoplasmic reticulum
DGAT1: diacylglycerol acyltransferase 1
DGAT2: diacylglycerol acyltransferase 2
ADRP: adipose differentiation-related protein
ATGL: adipose triglyceride lipase
HSL: hormone sensitive lipase
MGL: monoacylglycerol lipase
CIDE: cell death-inducing DNA fragmentation factor alpha (DFFA)-like effector
ABHD5: αβ hydrolase domain containing 5
HIG2: hypoxia-inducible gene 2
FASN: fatty acid synthetase
FFA: free fatty acids
ROS: reactive oxygen species
ATP: adenosine triphosphate
cAMP: cyclic adenosine monophosphate
TME: tumor microenvironment
HIF: hypoxia inducible factor
PKA: protein kinase A
AMPK: AMP-activated protein kinase
TAG: triacylglycerol
DAG: diacylglycerol
CE: cholesteryl ester
ccRCC: clear cell renal cell carcinoma
Pca: prostate cancer
ADT: androgen deprivation therapy
CRC: colorectal cancer
HCC: hepatocellular carcinoma
TNBC: triple-negative breast cancer
GC: gastric cancer
NSCLC: non-small cell lung carcinoma
